# The association between expressive language skills and adaptive behavior in individuals with Down syndrome

**DOI:** 10.1038/s41598-022-24478-x

**Published:** 2022-11-21

**Authors:** Laura del Hoyo Soriano, Jennifer Catalina Villarreal, Audra Sterling, Jamie Edgin, Elizabeth Berry-Kravis, Debra R. Hamilton, Angela John Thurman, Leonard Abbeduto

**Affiliations:** 1 MIND Institute, University of California Davis, 2825 50Th Street, Sacramento, CA USA; 2 Department of Psychiatry and Behavioral Sciences, Davis Health, University of California, Sacramento, CA USA; 3 Waisman Center and Department of Communication Sciences and Disorders, University of Wisconsin-Madison, Madison, WI USA; 4 Department of Psychology, University of Arizona, Tucson, AZ USA; 5 Departments of Pediatrics, Neurological Sciences and Biochemistry, Rush University Medical Center, Chicago, IL USA; 6 Department of Human Genetics, Emory University School of Medicine, Atlanta, GA USA

**Keywords:** Genetics, Psychology, Human behaviour, Quality of life

## Abstract

The primary goal of this study was to determine whether expressive language skills contribute to adaptive behavior (e.g., socialization and daily living skills) in children, adolescents, and young adults with Down syndrome (DS) whilst controlling for age and nonverbal cognitive ability. Expressive language was assessed using the psychometrically validated Expressive Language Sampling (ELS) conversation and narration procedures. The language produced was transcribed and analyzed to yield measures of expressive vocabulary, syntax, and intelligibility. Socialization and daily living skills of participants with DS were measured with the Vineland Adaptive Behavior Scales, 2nd edition (VABS-2) parent/caregiver rating form. Our results show that the three ELS measures were significantly correlated with multiple measures from the VABS-2 when controlling for age. Several correlations remained significant even when nonverbal cognitive ability was included as a control variable. Our results suggest that expressive language skills contribute to adaptive behavior in children, adolescents, and young adults with DS regardless of age and some of these associations are not explained solely by overall cognitive delays. Further studies including longitudinal data are needed to extend our results.

## Introduction

Down syndrome (DS) is the leading genetic cause of intellectual disability (ID), with approximately 1 in 691 live births affected^1^. The syndrome is typically caused by an extra copy of all or part of chromosome 21^2^, which results in a complex condition that affects both physical (e.g., facial dysmorphology, a disproportionately large tongue) and cognitive development^3^. Indeed, DS is associated with prototypical pattern of cognitive and behavioral strengths and challenges. More specifically, when compared either to individuals with typical development (TD) or with other neurodevelopmental disabilities (NDDs) of similar mental ages (MAs), individuals with DS demonstrate, as a group, relative strengths in the areas of nonverbal communication, gross motor skills, visual motor integration, and visual imitation^4,5^. In contrast, auditory short-term memory, episodic memory, aspects of visuospatial construction and executive function, and language are areas that are particularly challenging for individuals with DS^6–12^.

Language skills constitute one of the most impaired aspects of the cognitive profile of individuals with DS. Research has also shown, however, that within the area of language some aspects are generally more affected than others among those with DS^9^. For example, receptive language is usually an area of relative strength compared to expressive language skills (i.e., syntax, vocabulary, phonology). Indeed, there is considerable research documenting that multiple areas of expressive language lag behind MA expectations^13–15^. For example, individuals with DS score less well on measures of vocabulary and syntax when compared to younger TD peers^10,16–19^ and similarly-aged peers with other developmental disabilities, both matched on MA^17,20,21^. There is also evidence suggesting that expressive syntax skills are more impaired than are expressive vocabulary skills in individuals with DS^22^ as expressive syntax appears to lag relative to both nonverbal cognition and vocabulary level-expectations^13–15,23,24^. Therefore, severity of impairment also varies across different areas of expressive language in DS. In addition, hearing loss and differences in oral-facial anatomy also negatively impact speech perception and production in individuals with DS^25^, contributing to high rates of unintelligible speech. Even areas of relative strength are delayed relative to expectations based on chronological age (CA). Nonetheless, there is considerable heterogeneity in terms of level and profile of strengths and weaknesses among individuals with DS^26,27^.

Adaptive behavior is the collection of conceptual, social, and practical skills that are learned through experiences and are essential to meeting the demands of everyday life^28^. Consistent with the definition of ID, adaptive behavior is also an area of challenge for individuals with DS^29^. That is, ID is, by definition, characterized by deficits in cognitive function that would impact how the individual functions in their everyday life and, therefore, cognitive deficits are highly associated with difficulties in adaptive behavior. Interestingly, previous research in DS using the Vineland Adaptive Behavior Scales (VABS)^30^ has shown that children and adolescents may demonstrate better adaptive behavior performance, on average, in the domain of socialization than in the domain of daily living skills^29,31,32^.

Language skills are an important factor associated with the level of adaptive behavior achieved by individuals with DS. Indeed, language challenges appear to be a substantial barrier to independent functioning and meaningful inclusion in the community for individuals with DS^16^. A study of adolescents and young adults with DS^33^ documented associations between expressive and receptive language and adaptive behavior in functional skill areas such as communication, community use, and functional academics, as well as in the overall score for adaptive behavior as measured with the Adaptive Behavior Assessment System, Second Edition (ABAS-2)^34^. In fact, among other cognitive constructs evaluated (e.g., attention, memory, executive function), language was the skill most closely related to higher competence in overall adaptive skills for participants with DS^33^. It also has been found that the speech scale (which assesses articulation skills and phonology) of the parent-report Children's Communication Checklist, Second Edition (CCC-2)^35^, which assesses receptive and expressive language (speech, syntax, semantics, and coherence) and pragmatics, accounted for variance in adaptive behavior assessed with the ABAS-2 in adolescents with DS^36^. The close association between language, particularly expressive language, and daily functioning has also been documented for other developmental disabilities such as fragile X syndrome (FXS)^37^.

Because expressive language is one of the most impaired aspects of the cognitive profile of individuals with DS, and consists of diverse but related constructs (e.g., syntax, vocabulary, intelligibility) affected at variable levels in DS and possibly differentially allied to different dimensions of adaptive behavior, exploring these concurrent relationships is of special interest in this population. Examining the relationships between specific aspects of adaptive behavior (i.e., socialization and daily living skills) and specific aspects of expressive language (e.g., syntax, vocabulary, intelligibility) can help identify the language skills to be targeted in interventions designed to support greater independent functioning in those with DS. The aim of the present study was to examine these concurrent relationships for children, adolescents, and young adults with DS. In doing so, we assessed dimensions of spoken language (i.e., syntax, vocabulary, intelligibility) likely to be related to socialization (i.e., interpersonal relationships, play and leisure time, coping skills) and daily living skills (i.e., personal care, domestic skills, and community use).

We measured expressive language using a set of expressive language sampling (ELS) procedures, which have been psychometrically validated for use in DS^38^ and in other NDDs such as FXS^37^. ELS procedures entail collecting and analyzing relatively brief samples of spoken language in a naturalistic contexts that are representative of the individual’s “everyday” language activities^37^. The particular ELS procedures we used have been standardized to ensure consistency of measurements across individuals and occasions of assessment^37^. Importantly, there are multiple advantages to using ELS, compared to typical standardized assessments of language skills, when considering the link between expressive language and adaptive behavior in DS. For example, ELS procedures (1) use a format more closely aligned with real-world contexts and, therefore, are more likely to generalize to activities that are functional and meaningful for the participant^39^; (2) can yield multiple dependent variables, reflecting different domains of skill, that can be examined separately; (3) are less prone to noncompliance and floor effects as compared to standardized tests; and (4) can be collected quickly and often with minimal training of examiners, making ELS especially attractive for multi-site studies^38,40^.

The research questions and hypotheses of the current study were:

1. Are expressive language skills related to concurrent levels of adaptive behavior in the domains of daily living skills and socialization in children, adolescents and young adults with DS whilst controlling for CA? It was hypothesized that stronger expressive language skills, as assessed through the ELS procedures, would be associated with greater levels of adaptive behavior regardless of age. Such statistical control was needed given the anticipated correlation between age and expressive language skill, with the latter improving with age even among individuals with DS.
2. Are expressive language skills associated with specific aspects of daily living and socialization skills whilst controlling for age and nonverbal cognitive ability in children, adolescents, and young adults with DS? It was hypothesized that expressive language skills would be associated with specific socialization and daily living skills even after controlling for both CA and nonverbal cognitive ability. Such statistical control was needed given the anticipated correlation of nonverbal cognition and expressive language skill, with the latter improving with increases in cognitive ability in individuals with DS, as in individuals with TD.

## Methods

The data for the current study were collected as part of a larger multi-site study evaluating the psychometric properties of variables derived from ELS procedures^37,38^. Study procedures were reviewed and approved by Institutional Review Boards at all participating university sites located in Arizona, Georgia, California, and Wisconsin. Written informed consent was obtained from caregivers, and verbal assent was obtained from the youth with DS prior to beginning study procedures. The authors affirm that all procedures contributing to this work comply with the ethical standards of the relevant national and international committees on human experimentation and with the Helsinki Declaration of 1975, as revised in 2008. All data for the present study were collected at each participant’s initial annual visit.

### Data source and study sample

A total of 95 participants with DS between the ages of 7 and 23 years were included in the current study. The same inclusion criteria were followed in the current study as in the larger project. All participants provided medical documentation of DS (i.e., trisomy 21 or translocation) without mosaicism and met IQ criteria for ID (IQ ≤ 70). Based on parental report, the participant with DS also had to use speech as the primary mode of communication, with English as their primary language given that the ELS procedures were at that time available only in English. In addition, the participant could not have serious (uncorrected) visual and/or hearing impairments and could not be enrolled in a randomized controlled clinical trial or experience changes in medication, treatment, or educational interventions/programs within the 8 weeks prior to the initial visit. Participants who were non-compliant with the two ELS procedures used were excluded from the current analyses (see below for operationalization of compliance). Thus, a total of 10 participants (3 non-compliant in conversation, 6 in narration and 1 in both procedures) were excluded from the present analyses. Characteristics of participating youth with DS are described in Table 1.

**Table 1:** Characteristics of participating youth with DS (n = 95).

Measure	M	SD	Range
Chronological age (years)	15.89	4.92	7.05–23.72
SB-5 NV fluid reasoning raw score	10.63	4.55	1–23
SB-5 nonverbal deviation IQ	40.62	12.99	7.43–73.03
**Distribution of participants**			
Race	Ethnicity	Sex	Family Income
African American -7	Hispanic/Latinx -20	Female -50	USD < 25,000 -5
Asian Pacific islander- 2	Non-Hisp/Latinx- 75	Male -45	USD 25,000–50,000 -20
White -61			
Multiracial -6			USD 50,001–75,000 -14
Other- 3			USD 75,001–100,000 -16
Not reported- 16			
			USD 100,001–150,000 -20
			USD 150,001–550,000 -10
			USD > 250,001 -6
			Not reported- 14

### Measures

The measures reported here are a subset of a larger battery of direct assessments, questionnaires, and interviews from the project. The measures for the present study were the ELS procedures, the Stanford-Binet Intelligence Scales, Fifth Edition (SB-5) to assess nonverbal cognitive ability of participants, and the Vineland Adaptive Behavior Scales, Second Edition (VABS-2) to address socialization and daily living skills through parent/caregiver report.

### Expressive language sampling

Expressive language samples (ELS) were collected in two contexts—conversation and narration—from each participant. Conversation was always administered before narration, and each participant completed other measures between the two ELS procedures. Manuals describing ELS administration, training, and the assessment of fidelity are available at https://ctscassist.ucdmc.ucdavis.edu/ctscassist/surveys/?s=W9W99JLMNX.

The conversation consists of a 12-min interview-style interaction with a trained examiner. The examiner relies primarily on open-ended prompts to topics (e.g., “Tell me everything you did at school yesterday”) and broad follow-up questions and prompts (e.g., “What do you like about school?”) to encourage participant talk while minimizing their own talk. The examiner introduces predetermined topics in a standard order. The goal is to introduce at least three topics in addition to an initial idiosyncratic topic reflecting an interest of the participant (according to parent/caregiver report). Two alternate versions of the conversation task were administered, with different topics in each. Approximately half the participants received Version A and half Version B. Additional details of the conversation procedures can be found in Abbeduto et al.^37^

The narration consists of the participant telling the story depicted in a wordless picture book. The participant first looks at each page spread of the book without talking to gain a sense of the story. The participant then tells the story page by page, with the examiner controlling the page turning. The examiner’s prompts and responses are standardized and limited largely to the first page of the book. There is no set time limit for the narration task. Two books, each including 16 pages of story content from the Mercer Mayer’s “Frog” series, were used: Frog Goes to Dinner (Version A) and Frog on His Own (Version B). Although these books were created for children, they are actually rather sophisticated in the sense that they allow for a range of different types of language, form very concrete description of actions to explanation of character motivation, mental states, and emotions and have been used successfully even with TD adolescents and adults^41^. Approximately half the participants received Version A and half Version B. Non-compliance in the ELS procedures was defined as refusal to complete the procedures, no response, or repeated off-task behavior (e.g., saying, “I’m done” or refusing to talk) or, in conversation, failing to engage in talk for at least 9.5 min or, in narration, failing to produce relevant talk on at least 12 of the 16 pages in the book.

All ELS sessions were digitally audio recorded and analyzed using SALT: Systematic Analysis of Language Transcripts^42^. All transcripts were prepared by a primary transcriber and reviewed by a secondary transcriber before being finalized. Transcribers were blind to individual participant results for other measures. Talk was segmented into C-units, the upper bound of which is an independent clause and any modifiers. Inter-transcriber agreement was randomly assessed for 10 transcripts (4 Narration, 6 Conversations), with at least two from each site. In the larger study, inter-transcriber was 87% for utterance segmentation, 87% for identification of partly or fully unintelligible C-units, and 84% for identification of the exact lexical and morphemic content of each C-unit. In addition, inter-transcriber agreement was 76% for identification of the exact number of morphemes in each C-unit and 80% for the exact number of words in each C-unit^38^.

We focused on the three ELS outcome measures shown to have the strongest psychometric properties^37,38^. The measures were: (1) *Lexical Diversity*, which indexes the size of the participant’s expressive vocabulary and is operationalized as the number of different word roots in 50 complete and fully intelligible C-units (or the full sample of complete and fully intelligible C-units if the participant produces fewer than 50 C-units). Higher scores indicate more advanced expressive vocabulary. (2) *Syntax*, which indexed expressive syntactic complexity and is computed as the mean length of C-unit measured in morphemes (MLU) for complete and fully intelligible C-units. Higher scores indicate more advanced expressive syntax. (3) *Unintelligibility*, which is an index of speech articulation problems and is computed as the proportion of the total C-units that are either partly or fully unintelligible to the transcriber. Higher scores indicate more problems with articulation. A composite score for each measure was derived by first computing scores separately for conversation and narration tasks and then averaging performance across the two tasks. In the larger study, conversation and narration were readministered after 4 weeks to assess practice effects and test–retest reliability. Minimal practice effects were observed, and test–retest reliability was very strong for each of the 10 ELS measures for participants with DS, with the intraclass correlations ranging from 0.79 to 0.95 with all but one value above 0.83 and all *p* values > 0.005^38^. See Thurman et al. 2020 for detailed values. Note that each participant received the alternate versions of the conversation and narration materials in the two administrations, suggesting comparability of the versions. Importantly, comparability of version with a different sample of participants had been established previously^43^.

### Nonverbal cognitive ability

The Stanford-Binet Intelligence Scales, Fifth Edition (SB-5)^44^ was used to assess nonverbal cognitive ability. In our analyses, we used the Nonverbal Fluid Reasoning (NVFR) raw score derived from the Object Series/Matrices subtest as the control variable in assessing whether expressive language skills were associated with concurrent independent functioning. Note that we present the raw NVFR in Table 1. The deviation NVIQ is also derived from Object Series/Matrices subtest, indexing Nonverbal Fluid Reasoning (NVFR), and it is a z-score transformation based on the general population norms. The score is calculated following procedures outlined by Sansone and colleagues^45^ to avoid floor effects and obtain a normal distribution of the measurement for our sample of participants. This score is also presented in Table 1 for descriptive purposes. Note that we used NV raw scores rather than standard scores because interest was in the association between the absolute levels of ability achieved in expressive language and nonverbal cognition.

### Socialization and daily living skills

The Vineland Adaptive Behavior Scales-2nd edition (VABS-2)^46^ is a caregiver report measure of adaptive behavior. The measure was normed on individuals aged birth to 90 years, including individuals with ID. In the present study, we used raw scores for the three Socialization subdomains—Interpersonal Relationships (i.e., how the individual interacts with others), Play and Leisure (i.e., how the individual plays and uses leisure time), and Coping Skills (i.e., how the individual demonstrates responsibility and sensitivity to others)—as well as for the three Daily Living subdomains—Personal (i.e., how the individual manages tasks such as dressing and personal hygiene), Domestic (i.e., how the individual manages household tasks such as cleaning and laundry) and Community (i.e., how the individual manages time, money, technology, and employment opportunities). Raw scores range from 0 to near 130 depending on the subdomain, with higher scores reflecting greater ability. We focused on the subdomains rather than the superordinate domains of socialization and daily living because Shaffer et al.^47^ did not find any significant correlations of the superordinate domains with the ELS measures for their sample of participants with FXS. In addition, we were interested in evaluating the possibility that there are differential patterns of associations with the ELS measures and the specific adaptive skills represented in the various subdomains of the VABS-2. The VABS-2 has strong psychometric properties, including significant correlations with earlier versions of the VABS^48^ and the ABAS-2 and strong test–retest reliabilities for the six subdomains of interest in the norming sample (e.g., average correlations range between 0.76 and 0.92 across domains).

### Statistical analysis plan

First, descriptive statistics were computed for all variables of interest. We also examined the variables and their residuals for the assumptions of normality required in the parametric tests. Most VABS-2 variables violated assumptions of normality and transformations did not improve the distribution; thus, nonparametric correlations were implemented. To address the first research question, we computed partial Spearman correlations between each of the three ELS measures (derived from averaging the scores for conversation and narration) and each of the socialization and daily living skills raw scores while controlling for CA. To address the second research question, we computed nonparametric partial correlations between the ELS variables and the socialization and daily living skills raw scores while controlling for both CA and NV cognitive ability.

In each set of analyses, one-tailed inferential tests were used in all cases because we had clear hypotheses about the directionality of the relationships of interest. Familywise alpha levels for the correlations and for the partial correlations were maintained at *p* < 0.050 levels through application of Benjamini and Hochberg’s False Discovery Rate (FDR) procedures^49^.

### Ethical approval

This study was approved by the Institutional Review Boards (IRB) of each participating site: University of California Davis IRB Clinical Committee B (IRB403210), Education and Social/Behavioral Science IRB at the University of Wisconsin-Madison (IRB2013-0512), Rush University Medical Center IRB (IRB11112301), Emory University IRB (IRB00065271 UCDMC Language Study) and University of Arizona IRB (IRB1300000331). The authors assert that all procedures contributing to this work comply with the ethical standards of the relevant national and institutional committees on human experimentation and with the Helsinki Declaration of 1975, as revised in 2008. Informed consent was obtained from the parent or guardian of each participant before testing.

## Results

### Descriptive statistics

Means and standard deviations are presented for the ELS measures and the measures of adaptive behavior in Tables 2 and 3, respectively.

**Table 2:** Means and standard deviations for ELS measures.

Measure	Conversation		Narration		Combined^a^		N
	M	SD (range)	M	SD (range)	M	SD (range)	
Syntax	3.37	1.55 (1.03–6.78)	4.43	2.09 (1–8.76)	3.91	1.74 (1.16–7.22)	95
Lexical diversity	73.76	33.53 (10–154)	62.21	35.13 (1–141)	68.27	32.45 (7.5–135)	95
Unintelligibility	0.25	0.19 (0.02–0.77)	0.23	0.21 (0–0.78)	0.24	0.18 (0.01–0.7)	95

n = 95; ^a^Average of conversation plus narration.

**Table 3:** Means and standard deviations for VABS-2 measures.

Measure	Raw Score		Standardized Score		N
	M	SD	M	SD	
Interpersonal relationships subdomain	60.24	8.604	10.75	4.706	89
Play and leisure subdomain	46.25	10.274	9.95	4.321	91
Coping skills subdomain	40.22	11.161	11.48	2.259	82
Personal subdomain	65.52	11.837	9.73	3.662	92
Domestic subdomain	26.87	9.896	10.63	4.938	93
Community use subdomain	45.14	15.943	8.95	6.334	86
Socialization domain	–	–	72.25	14.27	80
Daily living skills domain	–	–	67.61	11.14	84
Adaptive behavior composite	–	–	71.89	27.32	72

*Note that missing values were due to parents not completing certain questions on each specific VABS-2 subdomain.

### Primary analyses

As seen in Table 4, nearly all the ELS measures were significantly correlated with the functional measures of adaptive behavior while controlling for CA, even after applying the FDR, with better performance on the ELS measures associated with greater levels of socialization and daily living skills. As seen in Table 4, correlations with the Personal and Domestic subdomains were weak. In contrast, ELS measures showed strong correlations with Interpersonal Relationships, Coping, and Community Use subdomains. None of the ELS measures were correlated significantly with the Play and Leisure subdomain raw score on the VABS-2. In addition, the correlation between ELS Unintelligibility and the VABS-2 Interpersonal Relationships subdomain just failed to reach significance (*p* = 0.051).

**Table 4:** Nonparametric partial correlations between ELS and VABS-2 raw scores controlling for chronological age.

Measures	VABS-2	VABS-2	VABS-2	VABS-2	VABS-2	VABS-2
	Interpersonal relationships	Play and leisure	Coping	Personal	Domestic	Community use
Syntax	0.33***	0.17	0.42****	0.26*	0.23*	0.51****
df	85	88	79	89	90	83
Lexical Diversity	0.39****	0.05	0.46****	0.21*	0.23*	0.54****
df	85	88	79	89	90	83
Unintelligibility	− 0.21	− 0.13	− 0.30**	− 0.23*	− 0.25**	− 0.37****
df	85	88	79	89	90	83

*p ≤ 0.050, **p ≤ 0.010, ***p ≤ 0.005, ****p ≤ 0.001, *****p ≤ 0.0005. Missing values were due to parents not completing certain questions on each specific VABS-2 subdomain.

Values in bold are significant at *p* ≤ 0.05 after FDR correction.

Partial correlations between the ELS and selected measures of adaptive behavior controlling for both CA and nonverbal ability (SB-5 NVFR raw score) are presented in Table 5. When including the SB-5 NVFR as a control variable along with CA, fewer correlations reached statistical significance in comparison to those represented in Table 4. In particular, after application of FDR, Syntax and Lexical Diversity were both still significantly correlated with raw scores on the Interpersonal Relationships (moderate to strong), the Coping Skills (strong), and Community Use (moderate to strong) subdomains, whereas Unintelligibility was significantly correlated with Coping skills and Community Use only (mild to moderate). Finally, raw scores on the Play and Leisure, Personal, and Domestic VABS-2 subscales were not correlated with any of the ELS measures.

**Table 5:** Nonparametric partial correlations between ELS and VABS-2 raw scores controlling for chronological age and the SB-5 NVFR raw score.

Measures	VABS-2	VABS-2	VABS-2	VABS-2	VABS-2	VABS-2
	Interpersonal relationships	Play and leisure	Coping	Personal	Domestic	Community use
Syntax	0.29**	0.11	0.35****	0.20	0.17	0.37****
df	83	86	77	87	88	81
Lexical Diversity	0.35****	0.11	0.40****	0.16	0.15	0.39****
df	83	86	77	87	88	81
Unintelligibility	− 0.18	− 0.08	− 0.26*	− 0.20	− 0.22*	− 0.31***
df	83	86	77	87	88	81

*p ≤ 0.050, **p ≤ 0.010, ***p ≤ 0.005, ****p ≤ 0.001, *****p ≤ 0.0005. Missing values were due to parents not completing certain questions on each specific VABS-2 subdomain.

Values in bold are significant at *p* ≤ 0.05 after FDR correction.

## Discussion

The goal of this study was to further understand the relationship between components of expressive language skill and specific aspects of adaptive behavior in children, adolescents, and young adults with DS whilst controlling for CA and NV cognitive ability. We found that all but 4 of the 18 bivariate correlations between the ELS measures and the parent/caregiver-reported measures of adaptive behavior were significant when controlling for CA. This pattern of findings is consistent with previous research in DS showing that more advanced language skills are related with more functional adaptive behaviors^33,36,50^, such as advanced skills for social and daily living functioning. The relationships among some of the constructs of interest, however, could be bidirectional. For example, more frequent and closer interpersonal relationships could create opportunities for practicing and acquiring new expressive language skills. The concurrent nature of the present data does not allow us to address these bidirectional possibilities. However, our data do establish the important link between expressive language abilities and the capacity for meaningful engagement in socialization and management of the tasks of daily living. This suggests a need for further research including longitudinal data.

Findings for our first research question indicate that limited expressive syntax and lexical diversity, as well as greater problems in intelligibility, are each associated with a less well-developed capacity for interpersonal relations, coping skills and daily living skills in children, adolescents, and young adults with DS. Interestingly, each of the expressive language skills measured in our study (e.g., syntax, lexical diversity, and unintelligibility) is associated with each of the subdomains of daily living skills (e.g., personal, domestic and community use skills). Importantly, research shows that people with DS with higher levels of daily living skills are more likely to participate in open employment-related activities and to eventually acquire a job, which is in turn related to a better quality of life for the family^51^. Therefore, interventions in DS with a special emphasis on expressive language should be considered as this could have a long-term impact on the level of independence achieved due to the highly verbal nature of the normative tasks of adulthood. With regards to the relationship between expressive language and socialization skills, there is a correlation with interpersonal relations and coping skills (or how the individual demonstrates responsibility and sensitivity to others). This pattern of findings may indicate that better expressive language skills promote social interactions and meaningful relationships or that greater socialization opportunities may facilitate development of expressive language skills. Note, however, that none of the ELS variables was related to play and leisure, which is surprising as previous research has shown a link between expressive language and play and leisure abilities in children with DS^52^, as well as in older adolescents and young adults with FXS^53^. In this regard, the ability to play and use leisure time in DS could be more closely aligned with other aspects of communication (i.e., pragmatics) than the structural language skills assessed in this study, which could be one of the reasons why we do not see any relationship between domains. It could also be that the association between expressive language and play and leisure skills is age-dependent in DS, and we have not observed such association in our sample as our age range was wide enough to control for CA given that we used raw scores in our analyses.

In terms of our second research question, we found that when controlling for CA and NV cognitive ability, far fewer correlations (8 out of the 18) remained significant. Specifically, only the links between ELS measures, Interpersonal Relationships, Coping and Community Use remained significant after controlling for NV cognition and age. These results suggest that specific expressive language skills make unique contributions to the prediction of specific aspects of adaptive functioning in individuals with DS over and above the contribution of nonverbal cognitive ability. It appears that expressive language skills are closely related to skills in the areas of interpersonal relationships, coping, and the use of community resources. Thus, how the individual participates in the community (e.g., how they use time, money, the telephone, public transportation, gain and maintain employment, or complete school tasks) might require expressive language skills to a greater degree than do domestic skills (e.g., cooking, laundry) and personal care skills (e.g., eating, dressing personal hygiene). These latter adaptive skills could be less verbal-dependent and more related to overall nonverbal cognition and/or other cognitive skills, which could be the reason why we do not see an association between these functional domains and our targeted expressive language constructs when controlling for NV cognition. For example, executive function skills may be more relevant than expressive language to follow cooking and laundry instructions. Memory skills are also necessary for personal hygiene (e.g., when was the last day I washed my hair, or whether I replaced the used towels for clean ones).

The observed specificity in the relationships between expressive language skills and adaptive behavior skills could be related to the nature of the adaptive behaviors targeted and the extent to which these adaptive behaviors require the specific expressive language skills assessed in individuals with DS. Interestingly, the specific pattern of relationships observed for individuals with DS differs from the patters observed for individuals with ID due to other causes. For example, Shaffer et al., did not find any link between the ELS measures of syntax and lexical diversity and the Socialization or Daily Living Skills domains but did find a link between unintelligible speech and the Daily Living Skills in individuals with FXS^54^. It is important to note, however, that Shaffer did not examine the subdomains but instead focused on the superordinate domains, which raises the possibility that there were also differential patterns of associations with the ELS measures were obscured by the use of the superordinate domain scores. Similarly, Abbeduto and colleagues^55^ found that lexical diversity, syntax, and unintelligibility were each related to all three Socialization subdomains, with the only exception being that unintelligibility was not linked to Play and Leisure in individuals with FXS. Abbeduto et al., however, did not examine the Daily Living subdomains. Taken together, these results suggest that the role that specific expressive language skills play in specific areas of adaptive behavior may be syndrome specific.

### Limitations

The current study has several limitations. First, it is important to be cautious about the generalizability of our results as we included only participants who were compliant on the administration of both conversation and narration, and for whom primary language was English. Second, although the inclusion of CA and NVFR as covariates controls for potential differences in participant characteristics, variability in samples across correlations (e.g., variable missing data in concrete VABS-2 subdomains) should be considered when interpreting our results. In addition, although the VABS-2 is available in multiple languages, additional work in terms of translation and cultural appropriateness is needed for the ELS procedures to be administered to participants for whom primary language is other than English. Importantly, this effort has begun for Spanish speakers^56,57^ but there is still a lot of work to do. Third, establishing a potential causal link between language and adaptive behavior will require simultaneous examination of both concurrent and longitudinal associations, as it is impossible to determine the direction of causality in the relationships observed through current cross-sectional analyses. In addition, it is important to note that we explored only a few possible dimensions of adaptive functioning as well as concrete expressive language constructs relative to structural language (e.g., we did not explore pragmatics). The relative contributions of language as well as other specific cognitive abilities to multiple dimensions of adaptive behaviors should be explored in future longitudinal research with an expanded range of measures of language (including receptive language, pragmatics, etc.), cognition, adaptive behavior, and several adaptive and functional skills relevant to independent functioning and inclusion in the community. We also recommend future studies stratifying analyses by age groups to better understand whether some of the targeted associations are age-dependent in DS.

## Conclusion

The present study has demonstrated that expressive language skills are linked to socialization and daily living skills in individuals with DS, which raises the possibility that interventions focused on improvements in expressive language may lead to, or at least provide a foundation for, improved adaptive behavior for those with DS. Unfortunately, there are few such evidence-based language interventions for this population focused on the targeted expressive language domains^58,59^. Therefore, there is a pressing need to develop such interventions. This study also addresses the call for psychometrically sound measures for evaluating treatment efficacy in studies of individuals with ID. In particular, we have shown previously that ELS-derived measures are feasible, are subject to only minimal practice effects, have strong test–retest reliability, and have construct validity for individuals with DS and other developmental disabilities^37,38,57^. The data from the present study show that differences on ELS-derived measures are associated with real-world functional competence (i.e., socialization and daily living skills) in children, adolescents, and young adults with DS, which is an association valued by treatment-regulating bodies, such as the U.S. Food and Drug Administration (FDA), when deciding on the utility of an outcome measure for establishing treatment efficacy. Based on our results, we encourage additional study of the link between other commonly used outcome measures in NDDs (e.g., clinician or parent ratings of perceived improvement, eye-tracking, computerized tests, etc.) and meaningful functional outcomes to support selection of functionally relevant efficacy endpoints for clinical trials in these populations.

## Data Availability

The datasets used and/or analyzed for the present paper can be made available upon a reasonable request to the corresponding author.
